# Role and Efficacy of 18F-FDG PET/CT in Following Up Desmoplastic Small Round Cell Tumor of the Abdomen: A Case Report

**DOI:** 10.7759/cureus.72209

**Published:** 2024-10-23

**Authors:** Juncheng Huang, Jonathan Chng, Hoi Yin Loi

**Affiliations:** 1 Radiology, National University Health System, SIngapore, SGP; 2 Pathology, National University Health System, Singapore, SGP; 3 Radiology, National University Health System, Singapore, SGP

**Keywords:** 18f-fdg pet/ct, desmoplastic small round cell, high-grade, metastatic malignancy, peritoneal mass

## Abstract

Desmoplastic small round cell tumor (DSRCT) is an exceptionally rare and highly aggressive primary peritoneal neoplasm predominantly seen in adolescents and young adults. Despite the availability of various treatment modalities, the prognosis remains grim due to the tumor’s aggressive nature and tendency for widespread metastasis. Although imaging of DSRCT is frequently documented using computed tomography (CT), the role of 18F-fluorodeoxyglucose positron emission tomography/computed tomography (18F-FDG PET/CT) in the initial diagnosis, staging, and particularly in the follow-up evaluation, is less explored. This case report details the role and efficacy of 18F-FDG PET/CT in the follow-up of a patient with extensive peritoneal involvement by DSRCT, demonstrating its added advantage in monitoring treatment response over conventional CT imaging.

## Introduction

Desmoplastic small round cell tumor (DSRCT) is an aggressive malignancy that arises predominantly within the peritoneum, characterized by the presence of small, round, and blue tumor cells surrounded by dense desmoplastic stroma [1]. The tumor primarily affects adolescent males and young adults, with a male-to-female ratio of 4:1 [2]. DSRCT is associated with a translocation between chromosomes 11 and 22, specifically t(11;22)(p13;q12), leading to the fusion of the EWSR1 and WT1 genes [3]. This genetic anomaly is believed to drive the oncogenesis of DSRCT, contributing to its aggressive behavior and poor prognosis [4]. We report a rare case of DSRCT in a young Asian male and highlight the added advantage of 18F-fluorodeoxyglucose positron emission tomography/computed tomography (18F-FDG PET/CT) over conventional CT imaging in the follow-up of his extensive peritoneal disease.

## Case presentation

A 19-year-old Asian male with no significant past medical history presented with a one-month history of persistent diarrhea, nausea, and unexplained weight loss. On physical examination, a large, firm, non-tender abdominal mass was palpated. Initial laboratory investigations, including complete blood count, renal and liver function tests, and tumor markers such as AFP, bHCG, CEA, LDH, and CA19-9, were unremarkable (Table 1).

**Table 1:** Laboratory findings on admission No evidence of raised tumor markers or inflammatory markers (e.g. white blood cells) was noted to suggest a clear etiology on the initial workup.

Parameters	Value	Reference Range	Unit
White Blood Cells	7.54	4.30 - 10.40	X 10^9/L
Red Blood Cells	4.67	4.50 - 5.75	X 10^12/L
Hemoglobin	13.2	13.1 - 16.8	g/dL
Platelets	415	150 - 410	X 10^9/L
Albumin, Serum	44	35 - 52	g/L
Bilirubin, Total	6	1 - 20	µmol/L
Alanine Transaminase (ALT)	29	6 - 40	U/L
Aspartate Transaminase (AST)	42	6 - 35	U/L
Alkaline Phosphatase (ALP)	84	45 - 150	U/L
Sodium	140	135 - 145	mmol/L
Potassium	4.4	3.5 - 5.2	mmol/L
Chloride	104	95 - 110	mmol/L
Bicarbonate	23	22 - 32	mmol/L
Urea, Serum	4.8	3.2 - 7.4	mmol/L
Creatinine, Serum	68	60 - 110	µmol/L
eGFR	>120	>90	ml/min/1.73m²
Alpha-Fetoprotein (AFP)	<2.0	0.9 - 8.8	µg/L
Beta-hCG, Serum (bHCG)	<3.0	≤5	IU/L
Carcinoembryonic Antigen (CEA)	<1.8	≤5.0	µg/L
Lactate Dehydrogenase (LDH)	193	120 - 250	U/L
Cancer Antigen 19-9 (CA 19-9)	<3	<37	U/mL

An initial abdominal radiograph was performed, which was unremarkable, failing to provide any clues to the underlying pathology. Given the palpable abdominal mass, a contrast CT scan of the abdomen and pelvis was subsequently ordered. The CT scan (performed in the portal venous phase) revealed multiple large peritoneal masses, some of which were conglomerated, occupying almost the entire abdominal and pelvic cavities. Additionally, there was evidence of hepatic metastasis, evidenced by several hypodense lesions seen throughout the liver parenchyma.

**Figure 1:**
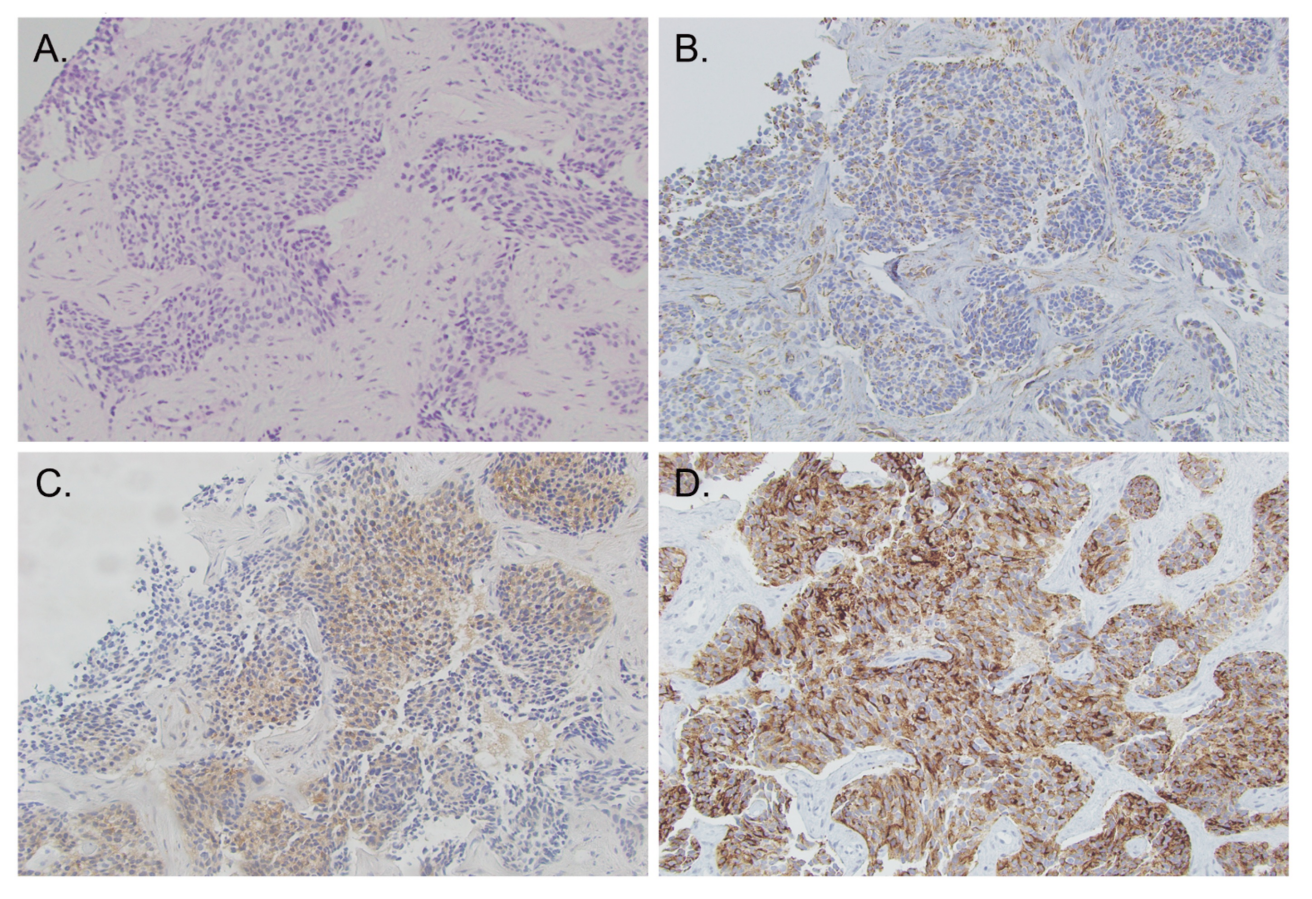
Histopathology of a desmoplastic small round cell tumor H&E staining (1A) revealed a high-grade tumor with undifferentiated nests of tumor cells surrounded by desmoplastic stroma, with morphology features suggestive of desmoplastic small round cell tumor. Immunohistochemical staining with vimentin (+) (1B), neuron-specific esterase (+) (1C), and epithelial membrane antigen (+) (1D) supports the diagnosis above.

A subsequent liver biopsy revealed the presence of a high-grade tumor with undifferentiated nests of tumor cells surrounded by desmoplastic stroma (Figure 1). The immunostaining profile was polyphenotypic with mixed cytokeratin, vimentin, and NSE staining of the tumor. These findings were supportive of DSRCT.

The patient was subsequently initiated on systemic chemotherapy. This consisted of three cycles of vincristine, doxorubicin, and cyclophosphamide, followed by two cycles of Ifosphamide, then one cycle of vincristine, actinomycin D, and cyclophosphamide, and finally one cycle of ifosfamide and etoposide.

**Figure 2:**
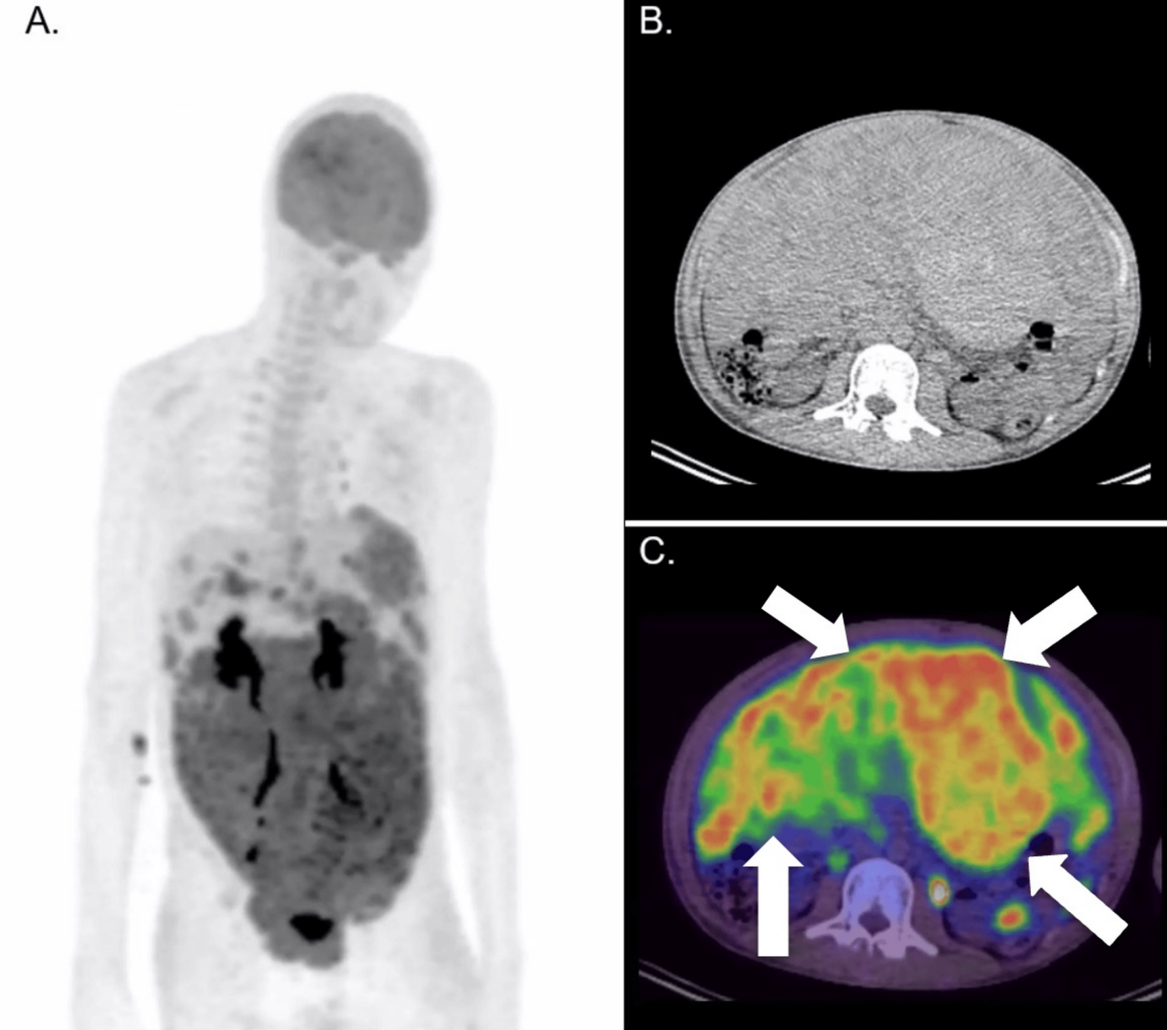
Pre-treatment 18F-FDG PET/CT scan The initial axial maximum intensity projection (MIP) (2A) and axial non-contrasted CT (2B) demonstrated extensive abdominal and pelvic masses, primarily involving the peritoneum and encasing most of the bowel loops. Fused PET/CT images (2C) highlighted intense FDG uptake in these masses, reflecting the high metabolic activity typical of DSRCT. The peritoneal and hepatic involvement was extensive (arrows), suggesting a significant tumor burden.

To further evaluate the extent of the disease and provide a baseline for future follow-up, an 18F-FDG PET/CT scan was performed (Figure 2). The scan demonstrated extensive hypermetabolic activity corresponding to the peritoneal masses and hepatic lesions observed on the CT scan, confirming widespread metastatic disease. The PET/CT images provided a more detailed and comprehensive assessment of the disease’s extent, clearly depicting the diffuse involvement of the peritoneum and liver. Given the extensive disease burden, the prognosis was considered poor.

**Figure 3:**
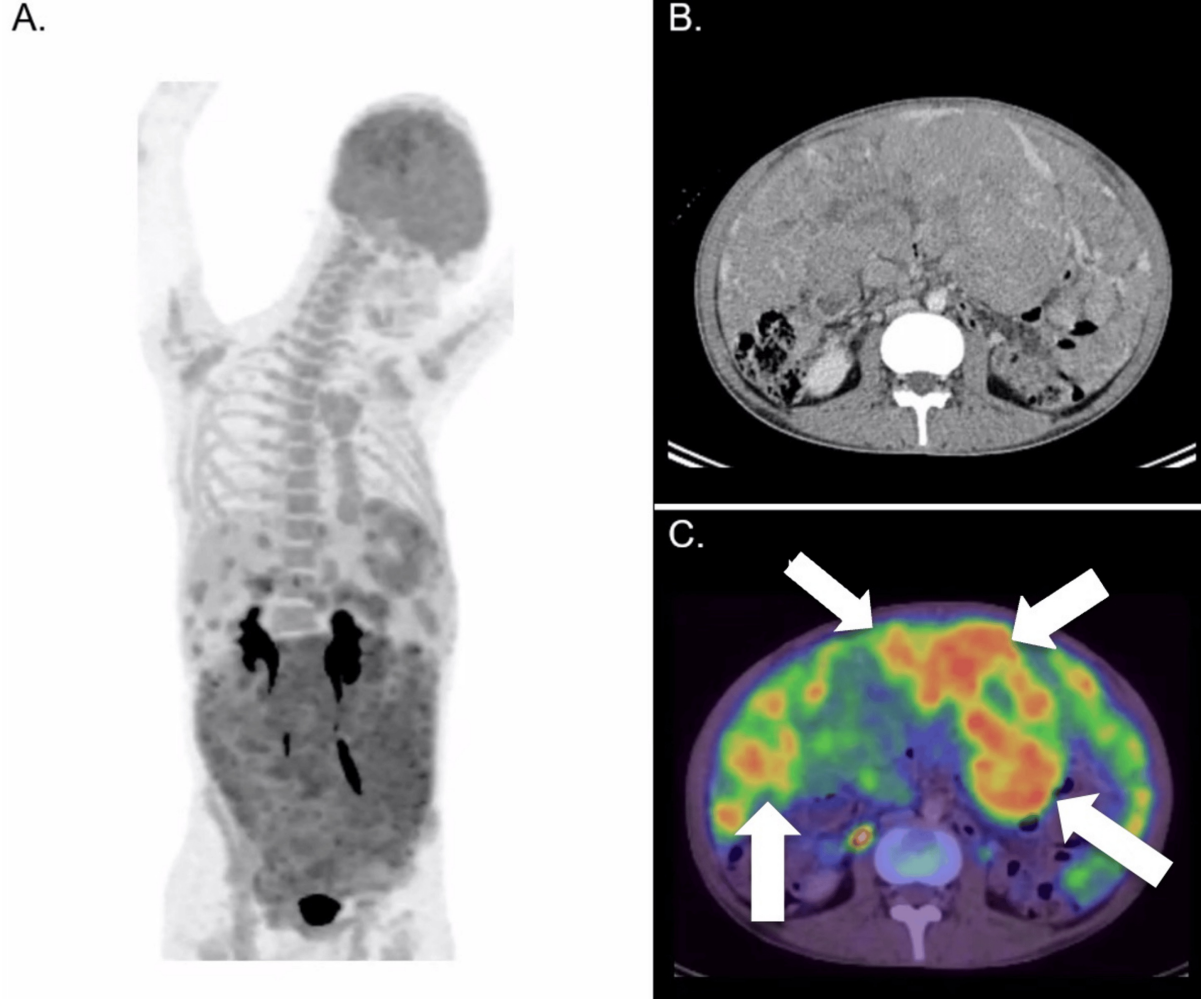
Follow-up 18F-FDG PET/CT scan (two months from the initial PET/CT scan) After two months of systemic chemotherapy, follow-up imaging was performed to assess the treatment response. MIP (3A), axial contrasted CT (3B), and fused PET/CT images (3C) were acquired. The slight reduction in the size of the peritoneal and hepatic masses was challenging to appreciate on follow-up CT images. However, the fused PET/CT images more easily demonstrated a decrease in metabolic volume (arrows), indicating a partial metabolic response to the therapy. 18F-FDG PET/CT: 18F-fluorodeoxyglucose positron emission tomography/computed tomography; MIP: maximum intensity projection

The follow-up PET/CT scan performed two months later (Figure 3) revealed an appreciable reduction in metabolic volume on the fused PET-CT images, despite only a slight reduction in tumor bulk on the non-contrasted CT (the metabolic tumor volume was computed using the 41% SUVmax method). Of note, the metabolic tumor volume reduced from a value of 12544 cm³ to 6080 cm³ (more than 50% reduction). The SUVmax had no significant reduction (from a value of 10.2 to 9.3). These favorable findings from the PET/CT scan provided confidence for a multidisciplinary team decision to recommend continuing the planned chemotherapy regime.

## Discussion

We present a case of a 19-year-old Asian male who presented with a history of persistent non-specific symptoms of diarrhea, nausea, and unexplained weight loss, with unremarkable initial physical examination and laboratory investigations. Contrast-enhanced CT revealed multiple large peritoneal masses and hepatic metastasis, of which the subsequent 18F-FDG PET/CT studies better demonstrated the disease extent and response to chemotherapy.

Given its rarity, DSRCT often presents a diagnostic challenge, frequently diagnosed at an advanced stage due to its nonspecific symptoms such as abdominal pain, distension, nausea, and diarrhea [5]. Imaging plays a critical role in the diagnosis, staging, and monitoring of DSRCT [6]. While conventional imaging modalities, such as CT and magnetic resonance imaging (MRI), are commonly used, they often fall short of accurately assessing the full extent of the disease, particularly in the presence of extensive peritoneal involvement.

18F-FDG PET/CT combines metabolic and anatomical imaging, offering a more comprehensive evaluation of malignant diseases. In DSRCT, this modality can provide additional information that might not be captured by CT alone, particularly in assessing the metabolic activity of the tumor, the extent of metastatic disease, and the response to treatment. However, the role of 18F-FDG PET/CT in the follow-up of DSRCT remains under-reported in the literature [7].

This case highlights the potentially critical role of 18F-FDG PET/CT in the management of DSRCT, particularly in the follow-up of patients undergoing treatment [8]. In this case, the initial PET/CT scan was instrumental in better illustrating the diffuse involvement of the peritoneum and liver as compared to the initial CT scan. Moreover, the follow-up PET/CT was effective, demonstrating the metabolic response to chemotherapy, compared to anatomical CT imaging alone which demonstrated a mild reduction in tumor extent.

To our knowledge, few studies specifically evaluating the efficacy of PET/CT in the follow-up of DSRCT of the abdomen have been studied in the pediatric population [9], with only one case report in the adult population published [10]. This case report shared how a 43-year-old man initially presented with retroperitoneal and mesenteric lymphadenopathy, which was diagnosed as a metastatic desmoplastic small round cell tumor (DSRCT). This was based on an initial biopsy and histology from subsequent surgery (tumoral radical resection, partial sigmoidectomy, and primitive iliac and periaortic lymphadenectomy). The patient experienced a relapse four years later and was commenced on chemotherapy. Throughout the chemotherapy regime, a series of 18F-FDG PET/CT scans were performed, showing progressive improvement, with remission after two years of treatment. Unfortunately, follow-up PET/CT scans later revealed a few retroperitoneal lymph nodes with pathological FDG uptake, though only measuring up to one centimeter in maximal diameter. These findings were suspicious for disease recurrence, and this prompted the clinical team to adjust the treatment plan and re-initiate the appropriate chemotherapy regimen. This case highlights the importance of 18F-FDG PET/CT in both the assessment of treatment response and the early detection of disease recurrence in cases of remission.

The use of 18F-FDG PET/CT in the follow-up of DSRCT patients is a promising modality to provide critical insights into the effectiveness of treatment, allowing for more informed decisions regarding whether to continue or modify ongoing treatment regimes, with the hope of improving outcomes in this otherwise dismal disease.

## Conclusions

This case underscores the importance of incorporating 18F-FDG PET/CT into the follow-up of patients with DSRCT, especially those with extensive peritoneal involvement. This imaging modality offers added imaging capabilities, providing detailed insights into the metabolic activity of the tumor and potentially improving the assessment of disease progression and treatment response compared to CT alone. As demonstrated in this case, 18F-FDG PET/CT can play a pivotal role in following up DSRCT, through enhancing confidence in response assessment and potentially helping tailor treatment strategies to improve patient outcomes.
